# Factors influencing the protective behavior of individuals during COVID-19: a transnational survey

**DOI:** 10.1038/s41598-021-01239-w

**Published:** 2021-11-04

**Authors:** Chia-Chun Tang, Hsi Chen, Wei-Wen Wu

**Affiliations:** 1 School of Nursing, College of Medicine, National Taiwan University, Taipei, 10051 Taiwan; 2 Department of Nursing, National Taiwan University Hospital, Taipei, 10051 Taiwan

**Keywords:** Preventive medicine, Public health

## Abstract

To slow the spread of infectious disease, it is crucial to understand the engagement of protective behavior among individuals. The purpose of this study was to systematically examine individuals’ protective behaviors and the associated factors across countries during COVID**-**19. This causal-comparative study used a self-developed online survey to assess individuals’ level of engagement with six protective behaviors. Analysis of variance and McNemar’s test were employed for data analysis. Three hundred and eighty-four responses were analyzed. The majority of participants lived in three areas: Taiwan, Japan, and North America. Overall, the participants reported a high level of engagement in protective behaviors. However, engagement levels varied according to several demographic variables. Hand hygiene and cleaning/ventilation are two independent behaviors that differ from almost all other protective behaviors. There is a need to target the population at risk, which demonstrates low compliance. Different strategies are needed to promote specific protective behaviors.

## Introduction

The year 2020 had an unusual start with the outbreak of coronavirus disease 2019 (COVID-19), which was first noticed in Wuhan, China and quickly spread to 213 countries within 4 months^1^. In March 2020, the World Health Organization (WHO) announced that COVID-19 could be characterized as a pandemic. Almost 1 year later, more than one billion coronavirus cases have been confirmed with two million deaths^2,3^. For many people, their daily lives were severely disturbed by this devastating situation. They were forced to quickly respond to the crisis, which included adapting behaviors to protect themselves. While strong evidence has supported that individuals’ behavior is the key to slowing down the disease spread and reduce morbidity^4,5^, there is a pressing need to evaluate the current protective behaviors and the related factors in a time**-**dependent manner, across countries.

COVID-19 is transmitted from person to person mainly through close contact and larger respiratory droplets. Hence, in general, it is recommended that individuals wear masks, avoid traveling, maintain social distancing, observe cough etiquette, maintain environmental cleanliness, and practice hand hygiene. However, many factors play an important role in an individual’s protective behavior adoption. For instance, during H1N1 influenza, differences in demographics, knowledge, perceptions, risk-specific worries, communication inequalities, and trust in the source of information are associated with practicing protective behaviors^5^. Similarly, several studies focused on protective behaviors during the COVID-19 outbreak and suggested parallel findings: demographics^6,7^, residence^8^, and perceptions^9,10^ are significantly related to an individual’s adherence to protective measures. Other studies have indicated that personal hygiene^11^, efficacy^9^, and background of medical professionals^12^ may affect the adherence to protective measures.

However, this information regarding factors affecting the adoption of protective behaviors information is diverse, complicated, confined to specific areas, and lacks systematic integration, which adds to the difficulties in preventing disease spread through behavioral strategies. There is also a lack of studies exploring the relationship between protective behaviors. Although we know that health behaviors may correlate with each other^13^, it is unclear whether we can expect a person to perform a certain protective measure along with other measures, or, on the contrary, a certain behavior is not related to other protective behaviors. Furthermore, there is a paucity of transnational studies. Thus, the goal of this study was to systematically examine individual protective behaviors and associated factors internationally. The specific aims were to: (1) develop a survey based on health belief model (HBM) to investigate how individuals were adopting protective behaviors during the COVID-19 pandemic, (2) examine whether individuals with different demographic variables differ in the adoption of protective behavior, and (3) examine the relationship between a set of protective behaviors.

## Results

Six hundred and twenty-nine responses returned with one duplicate response, 240 incomplete responses, and four responses failing attention check items, leaving 384 effective responses in the final analysis. Of the 384 responders (mean age = 39.92, SD = 14.65), 106 (27.6%) were healthcare professionals. The majority lived in Taiwan (n = 258, 67.19%), followed by Japan (n = 86, 33.33%), North America (n = 31, 8.07%), Europe (n = 5, 1.3%), and China (n = 4, 1.04%). Most respondents were women (n = 238, 62%) and had completed higher education (i.e., bachelor/associate degree or above, n = 352, 91.67%). While 182 (47.4%) of the responders were married, 297 did not have children (77.3%). Sixty-five (16.93%) responders had chronic diseases. Table 1 shows the detailed demographic information.

**Table 1:** Demographic information (n = 384).

Variables	Mean/number	SD/percentage
Age	39.32	14.65
**Sex**		
Male	145	37.8
Female	283	62
Not specify	1	0.3
**Marital**		
Married	182	47.4
Single	177	46.1
Divorced	12	3.1
Live together	8	2.1
Widowed	2	0.5
Prefer not to say	3	0.8
**Having children**		
Yes	87	22.7
No	297	77.3
**Education**		
Primary school or lower	1	0.3
Junior and senior high school	31	8.1
College/University	209	54.4
Graduate school	143	37.2
**Having chronic disease**		
Yes	65	16.9
No	319	83.1
**Healthcare professionals**		
Yes	106	27.6
No	278	72.4
**Places of residence in the past 6 months**		
Taiwan	258	67.2
Japan	86	22.4
North America	31	8.1
Others	9	2.3

### Frequency of engaging in protective behaviors (Table 2)

**Table 2:** Post hoc (Scheffe’s test) results: the differences of protective behavior engagement according to demographic variables.

Behavior	Demographic variables								
	All responses (n = 384)				Taiwan (n = 258)				Japan (n = 86)
	Residence	Sex	Having children	Chronic disease	Health background	Sex	Having children	Chronic disease	Sex
Wearing facemask	Japan > Taiwan***	–	–	–	–	–	–	–	–
	Japan > NA*								
Avoid nonessential traveling	Japan > Taiwan ***	–	–	Yes > no*	–	–	–	–	–
	NA > Taiwan***								
Social distancing	Japan > Taiwan ***	–	–	–	Yes > no*	–	Yes > no**	–	–
	NA > Taiwan*								
Hand hygiene	Japan > Taiwan ***	Female > male*	–	-	–	Female > male*	Yes > no*	–	–
Cough etiquette	Japan > Taiwan*	Female > male*	–	–	–	–	–	–	Female > male*
Cleaning and ventilation	–	Female > male*	Yes > no*	–	Yes > no*	Female > male*	Yes > no*	No > yes*	–

NA = North America, – = There is no significant difference between groups, ***p < 0.001, **p < 0.01, *p < 0.05.

Overall, the participants reported a high level of engagement (i.e., often or always) of practicing each protective behavior. Most respondents indicated that they were often or always upholding cough etiquette (91.1%), wearing masks (70.1%), maintaining social distances (63.8%), washing hands (60.2%), avoiding travel (56.8%), and cleaning/ventilating environment (54.7%). Females engaged in more hand hygiene (*p* < 0.05), cough etiquette (*p* < 0.05), and cleaning and ventilation (*p* < 0.01) than males. People who had children, cleaned and ventilated their environment more often than those who did not (*p* < 0.05). People with chronic disease, traveled less than their healthy counterparts (*p* < 0.05).

When grouping individuals according to their residence, statistically significant results were obtained for most behaviors, including wearing facemasks (F (2,372) = 14.057, *p* < 0.001), avoiding traveling (F (2,372) = 10.161, *p* < 0.001), social distancing (Welch’s F (2, 77.973) = 21.532, *p* < 0.001), washing hands (F (2,372) = 9.333, *p* < . 001), and upholding cough etiquette (F (2,372) = 3.167, *p* < 0.05). Post hoc analysis (Scheffe’s test) revealed that people in Japan wore facemasks more frequently than those in Taiwan (*p* < 0.001) and North America (*p* < 0.05); people in Taiwan traveled more than people in Japan (*p* < 0.001) and North America (*p* < 0.001); people in Taiwan practiced social distancing less than people in Japan (*p* < 0.001), and in North America (*p* < 0.05). People in Taiwan observed less hand hygiene (*p* < 0.001) and cough etiquette (*p* < 0.05) than those in Japan. However, no statistically significant difference was found in terms of whether having a health-related profession background would have an influence on behavior.

To further analyze the influences of demographic variables in area-specific samples, we found that, in Taiwan, health professionals practiced more social distancing (*p* < 0.05), hand hygiene behavior (*p* = 0.10), and cleaning and ventilation (*p* < 0.05) than non-health professionals. Females engaged more frequently in hand hygiene (*p* < 0.05) and cleaning and ventilation (*p* < 0.05) than males. People with children had more social distancing (*p* < 0.01), hand hygiene (*p* < 0.05), and cleaning and ventilation (*p* < 0.05) than those who did not. People with chronic disease cleaned and ventilated environments less than those without (*p* < 0.05). The only statistically significant difference observed in the Japanese sample was sex and cough etiquette; females engaged more in cough etiquette than males (*p* < 0.05).

### Relationship between protective behaviors (Table 3)

**Table 3:** McNemar’s test results: relationship among protective behaviors.

	W	A	S	H	Co	C
Wearing facemask (W)	–	–	–	All***, T***, J**	J*	T*, J***
Avoid nonessential traveling (A)		–	–	All***, T*, J*	J*	T***, J***
Social distancing (S)			–	All***, T***, J***	All*	T**, J***
Hand hygiene (H)				–	All***, T***	All***, T***
Cough etiquette (Co)					–	T**, J*
Cleaning and ventilation (C)						–

All = all responses (including Taiwan, Japan, North America, and others, n = 384), Taiwan = Taiwan’s responses (n = 258), Japan = Japan’s responses (n = 86), ***p < 0.001, **p < 0.01, *p < 0.05.

The frequency of practicing each protective behavior were classified into lower and higher frequency groups, divided by the mean value of that behavior. Based on the results of McNemar’s test, statistically significant differences were found between hand hygiene and all other behaviors (*p* < 0.001) and between social distancing and cough etiquette (*p* < 0.05). When focusing on specific areas, hand hygiene and cleaning/ventilation were both significantly different from all other protective behaviors in Taiwan. The responses from Japan showed similar results: cleaning and ventilation were significantly different from all behaviors except for hand hygiene. Hand hygiene was also significantly different from most behaviors, including wearing facemasks, avoiding nonessential travel, and social distancing. In addition, cough etiquette was significantly different from wearing facemasks and avoiding nonessential travel.

## Discussion

This study was guided by the HBM to structurally survey a set of protective behaviors and their associated factors across countries, several months after the onset of the COVID-19 pandemic. The validity and reliability of the author-established survey were good to excellent^14^. The 384 effective responses were mainly obtained from three countries or areas: Taiwan, Japan, and North America. The majority of the participants were middle-aged and highly educated.

The first aim of this study was to describe how individuals adopt protective behaviors concerning different demographic variables. We found that most individuals often or always engaged in protective behaviors, especially over 90% of them practiced cough etiquette and over 70% wore masks frequently. This result echoes the findings of other COVID-19 studies, which revealed that 70–90% of people adopted recommended behavior in the United States, Europe, Qatar, Veniamin, and India^6,7,11,12,15–18^. That is, people were highly aware of and actively responded to the ongoing pandemic. However, our results revealed a lower adherence rate to hand washing than in almost all published studies. This difference may be due to the research period, while most relevant studies collected this information at the very beginning of the pandemic (March 2020) and observed an increasing trend of adopting protective measures over weeks^15,16^, our survey was conducted later, and suggested a slight decrease in the adoption of protective measures. A decreasing trend in the adoption of protective measures was also observed by Dohle et. al., who followed up their survey (first conducted in March) in May^6^. This reflects that, regardless of the ongoing pandemic, the engagement of protective measures fluctuates, and when a pandemic lasts over 3 months, “behavioral fatigue” may be an issue calling more discussions and interventions^19^. While face masks still earn a high level of engagement because of government regulations in many countries, authorities may want to pay more attention to consistently promoting hand washing.

The levels of protective behavior engagement were affected by several demographic variables, including residence, health-related background, sex, and having children. Specifically, our results revealed that people living in Taiwan were less likely to engage in protective behaviors than those living in Japan or North America. In addition to different government support^18^, this may be associated with different disease situations in these areas. During the data collection period, Taiwan was in a more stable situation with about 447 COVID cases (incidence rate < 0.0019%) compared to Japan (18,476 cases, incident rate < 0.015%) and North America (> 2.6 million cases, > 0.8% incident rate)^2^. It is assumed that people living in an area with a more severe situation adhere more to protective behaviors than those living in areas with relatively stable situations. Because it is difficult to detect potential factors affecting behaviors under the ceiling effect of high adherence rate, the slightly lower adherence rate in Taiwan actually gives us more room to explore possible relationships between demographic variables and behavior. For example, Jose et al.^17^ examined a group of people who highly adhered to protective behaviors and found no relationship between the level of behavioral engagement and if an individual has a health-related background, which is similar to our findings in a Japanese sample^17^. However, we observed significant differences in the frequencies of performing protective behaviors between the general population and individuals with health-related backgrounds. This suggests that while the general population may be alert to the pandemic and quickly change their behavior, the changes may last for only a limited period. On the other hand, individuals with a health-related background may sustain their behavioral changes longer. Future studies are needed to examine whether health-related backgrounds contribute to adoption of protective behavior.

Similar to the findings of many studies^6,7,9–11,15^, our results indicate that women are more likely to adopt protective behaviors. Although the reason for this is not clear, it may be associated with different beliefs. In their study, Wise et al. pointed out that ‘females thought that they were more likely to pass on the virus if infected than males could’^16^; Pagnini et al.’s findings also indicated that women were more worried about the pandemic^10^. Interestingly, based on the results from a study focused on the influenza pandemic, men tended to wear fewer masks but were more willing to be vaccinated compared to women^20^. To uncover why sex causes differences in behavior engagement is an important topic for future studies to target specific populations with lower compliance.

In line with another study^6^, it was observed that people who have children, follow protective measures more often and people with chronic disease travel less, and when caring for such vulnerable populations, one should be more careful about using protective measures. In contrast, a surprising finding about individuals with chronic disease was that they cleaned and ventilated environments significantly less than those without chronic disease in Taiwan. A similar situation was observed in the USA, where health status seems to be unrelated to protective behavior^15^. Another German study mentioned that health status has a small correlation with behavioral acceptance but is not associated with behavioral adoption^6^. Although there is no explanation for this phenomenon, some relevant information may be considered. For instance, based on our clinical experiences, some patients with cancer described that the environment is friendlier to them now, because more people are aware of disinfecting the environment. A study conducted in Italy indicated that physical status was not related to worry about the pandemic^10^. These clues suggest that some vulnerable people have a more relaxed attitude toward the pandemic and are worth investigating in order to protect the population at risk for infectious diseases.

In response to the second aim regarding the relationship among protective behaviors, it was found that hand hygiene and cleaning/ventilation are two independent behaviors that differ from almost all other protective behaviors regionally and internationally. This result stimulated considerations when promoting a set of protective measures to stop the spread of disease. It seems that the knowledge, perception, and decision making of practicing hand hygiene or cleaning/ventilation may differ from those of adopting other protective behaviors. Factors, such as different promotion strategies, advertisement, regulation, and evidence level of each protective measure may contribute to these differences. We certainly cannot expect individuals who wash their hands frequently also put on their masks. Authorities may want to provide sufficient information and enhance the motivation for each desired behavior separately. There are limitations to this study that should be considered. Similar to other online surveys, our population was young, with a high education level. This may raise concerns of collider bias^21^ and limit the generalizability of our results. However, our results were similar to other studies that have included samples with diverse educational background and age groups. For example, a German study (n = 3189) which ensured equal distribution of educational level and age group also suggested that female gender was associated with higher odds of adopting protective behavior^22^. On the other hand, such limitations control the possible effects of age and education. Future studies may want to include more diverse samples, especially those with lower education or socioeconomic status.

## Conclusion

This study investigated the status of the engagement of a set of protective measures and their relationship with each other and with demographic variables across different countries during the COVID-19 pandemic. The established survey can be utilized to investigate individuals’ behavior and perception of infectious diseases or public health events in the future. Our results showed the need to target a particular population that is at risk and demonstrates low compliance. It also suggests that authorities may want to design specific strategies for different protective behaviors and sustain the adoption of behavior during the ongoing pandemic. Future studies are needed to confirm the factors that contribute to a low compliance rate.

## Methods

This was a causal-comparative study^23^ with a cross-sectional design. The study was reviewed and approved by the research ethics committee of the National Taiwan University Hospital. All methods have been performed in accordance with the Declaration of Helsinki. Electronic informed consent was obtained from all participants.

### The survey

The survey was developed based on a literature review and the HBM model by the research team, consisting of professionals with nursing and psychological backgrounds. A couple of medical experts were invited to review the content validity and 30 individuals were recruited to fill out the questionnaire as a pilot. The results were analyzed using factor analysis to verify the construct validity. The finalized items were translated and back-translated to confirm the English and Japanese versions. Cronbach’s alpha ranged from 0.66 to 0.85 for all subscales in the main study. The final survey can be found in the supporting information.

In this study, we employed the survey to collect demographic data and assess the frequency of practicing protective behaviors using a five-point Likert-type scale. Specifically, we investigated six protective behaviors, which were suggested across countries during the COVID-19 pandemic^24–26^: wearing facemasks, avoiding nonessential travel, social distancing, hand hygiene, cough etiquette, and cleaning and ventilation.

### Sampling and survey distribution

The survey was advertised on social media (Facebook, Instagram) and search engine (Google) from June 8th 2020 to June 22nd 2020. The target population included individuals who lived in Taiwan, the United States, Canada, and Japan at the time when the study was conducted. Despite the 2-week advertising period, the survey was kept open for 3 weeks. Participants were required to be at least 20 years old and be able to read and understand the provided language in the survey.

### Statistical methods

Descriptive analysis was used to analyze the demographic data of the participants. Analysis of variance (ANOVA) was applied to test if the frequency of engaging in behaviors differed according to (1) residence, (2) health profession-related background, (3) sex, (4) having children or not, and (5) having chronic disease or not. Homogeneity of variance was tested by Levene’s test, and if variance was not homogeneous, Welch’s test was applied instead. McNemar’s test was used to analyze the relationship between protective behaviors.
